# Increased excitability of renal sensory neurons by vasopressin in male and female Sprague-Dawley rats

**DOI:** 10.1093/ajh/hpag095

**Published:** 2026-07-30

**Authors:** Sebastien Hayoz, Christopher T Banek

**Affiliations:** Department of Surgery, Division of Autonomic Neuromodulation, University of Minnesota, Minneapolis, MN, United States; Department of Surgery, Division of Autonomic Neuromodulation, University of Minnesota, Minneapolis, MN, United States

**Keywords:** vasopressin, renal nerves, dorsal root ganglion, sensory nerves, hypertension, polycystic kidney disease, patch clamp, electrophysiology

## Abstract

**Background:**

Preclinical models of cardiovascular and kidney diseases, such as hypertension and polycystic kidney disease (PKD), are associated with increased vasopressin (AVP) and increased renal afferent nerve activity (ARNA). We have also shown that ablation of renal sensory nerves can mitigate HTN and PKD progression in preclinical models. Yet, the underlying cause of elevated renal sensory nerve activity in disease remains unclear, and the role of vasopressin remains untested. Therefore, we hypothesized that AVP would increase the excitability of renal-specific dorsal root ganglia neurons (rDRGN) in both male and female rats.

**Methods:**

To address this hypothesis, rDRGN were isolated from both male and female Sprague-Dawley (SD) rats and cell excitability changes with AVP were measured by whole-cell patch clamp in current-clamp mode. Excitability was quantified by mean action potential (AP) frequency, rheobase, and multi-firing threshold. Dose-dependent responses to AVP (10^−12^-10^−6^ M) were measured and compared to vehicle (saline).

**Results:**

Cell excitability was increased by AVP in both male and female cells, increasing AP frequency and lowering rheobase and multi-firing threshold. The effective excitatory range of AVP was 10^−10^-10^−6^ M in female cells, and 10^−8^-10^−6^ M in males.

**Conclusions:**

These experiments support our initial hypothesis that AVP would directly increase rDRGN excitability. This excitatory effect may support a novel role through which peripheral sensory nerves interact with and respond to AVP. In turn, this AVP–ARNA interaction may contribute to elevated peripheral sensory nerve activity and disease progression where AVP is elevated, such as hypertension and PKD.

## Graphical abstract

**Graphical Abstract:**
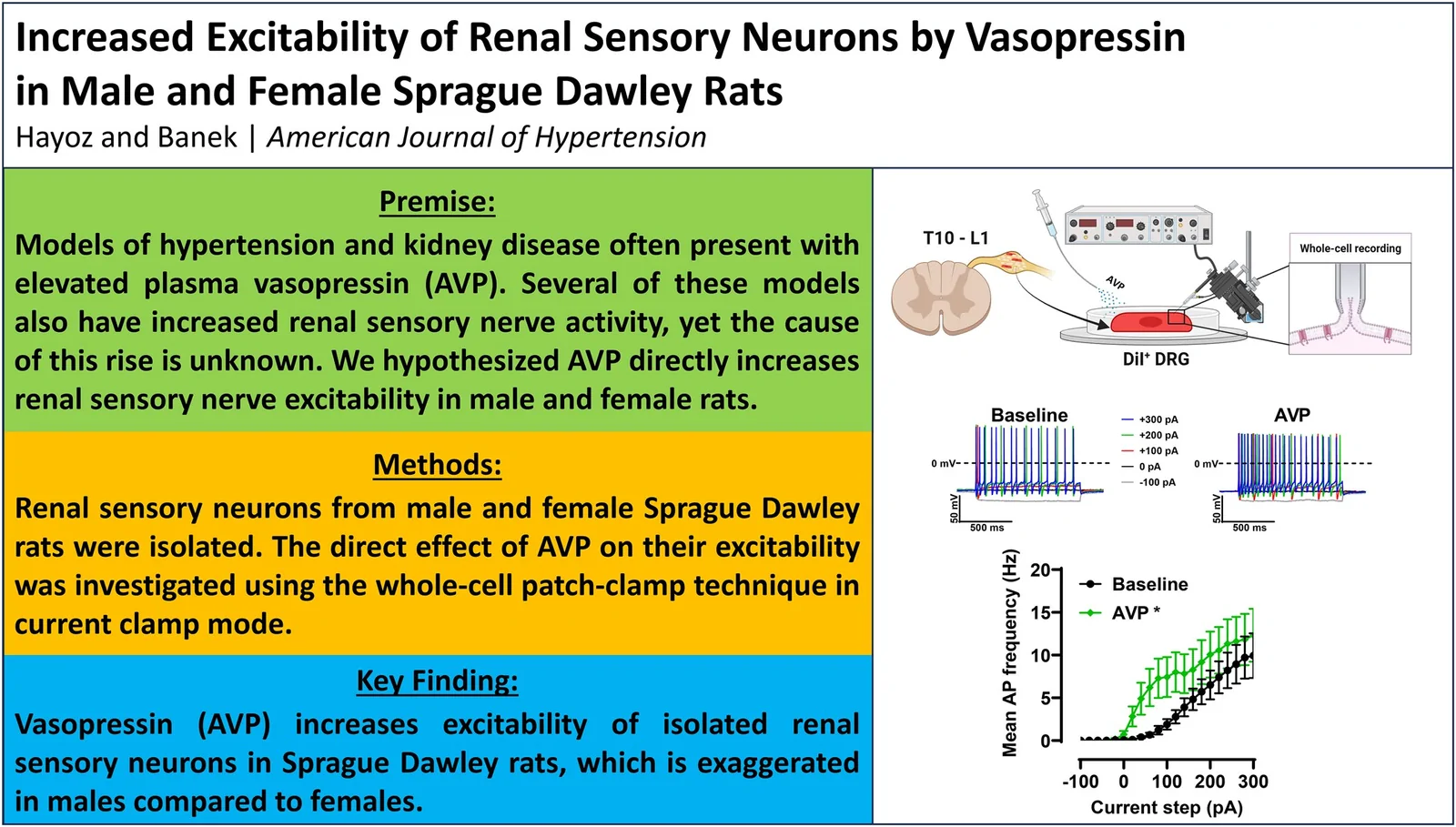

## Introduction

Our laboratory has demonstrated that afferent renal nerve activity (ARNA) is increased in models of hypertension and polycystic kidney disease (PKD), and ablation of renal nerves mitigates disease progression.^1–3^ However, the signal(s) driving this pathological sensory nerve activity remain unclear. There is evidence that vasopressin (AVP) action might be involved in ARNA. AVP directly modulates cyst progression in PCK rats, an animal model of PKD,^4^ and plasma AVP levels are higher in DOCA-salt hypertensive rats compared to control animals.^5^ Circulating AVP or other AVP biomarkers are elevated in patients suffering from hypertension^6^ and PKD.^7^ Although the direct effects of AVP on vascular and renal tissues are well described, its direct interaction with peripheral sensory neurons remains unclear. Based on the transcriptomic database from Ray and colleagues,^8^ dorsal root ganglion nerves (DRGNs) from humans and mice express AVP receptors (eg, V1a and V2). Therefore, we hypothesized that AVP drives increased excitability of renal afferent nerves. To test this hypothesis, we recorded the excitability of freshly isolated renal-specific dorsal root ganglia neurons (rDRGN) from male and female Sprague-Dawley (SD) rats and their response to AVP. The excitability of rDRGNs was quantified both in the absence and presence of AVP.

## Methods

### Animals and tissue collection

These experiments were completed at the University of Minnesota, approved by the Institutional Animal Care and Use Committee, and conducted in accordance with the National Institutes of Health Guide for the Care and Use of Laboratory Animals.^9^ All salts and chemicals were ordered from Sigma-Aldrich unless specified otherwise. Six days prior to rDRGN isolation, male and female SD rats (9-12 weeks old) were anesthetized (2%-3% isoflurane) to perform a laparotomy, and rDRGN were labeled by subcapsular injection of DiI [15 µL; 10 mg/mL in DMSO]). Rats were euthanized 6 days after DiI labeling, and dorsal root ganglia (DRG) were extracted from the T10-L1 segments of the spinal cord^10^ in dissection solution (132 mM NaCl; 5.3 mM KCl; 1.01 mM NaH_2_PO_4_; 10 mM HEPES; 5.5 mM glucose; 4 °C; pH 7.4). DRG were dissociated with DMEM F12 solution (Corning) containing 125 U/mL Collagenase XI (40 minutes; 37 °C; 10 rpm). Dissociated neurons were centrifuged and resuspended into 200 µL of dissection solution with 10% FBS, plated on poly-L-lysine-coated coverslips, and incubated overnight (37 °C; 20% O_2_; 5% CO_2_) in DMEM F12 (5% FBS and 1× penicillin-streptomycin). Neurons were used for patch-clamp electrophysiology within 24 hours post-isolation.

### Electrophysiological recording

Coverslips were transferred to the recording chamber at room temperature (Warner Instruments) mounted on the stage of an inverted microscope (Olympus), and bathed in extracellular solution (132 mM NaCl, 4 mM KCl, 1.2 MgCl_2_, 1.8 mM CaCl_2_, 10 mM HEPES, 5.5 mM glucose; pH 7.4; 296 mOsm). DiI-positive neurons were visualized and recordings were amplified, digitized, and recorded using an Axopatch 200B, Digidata 1550B, and pCLAMP (v.11.3) software (Molecular Devices). Fire-polished glass pipettes had a resistance of 4-7 MΩ when filled with pipette solution (10 mM NaCl; 130 mM KF; 4.5 mM MgCl2; 10 mM HEPES; 9 mM EGTA; pH 7.4; 312 mOsm). Following establishment of a whole-cell seal, the membrane test utility was used to record cell capacitance and access resistance values. Neurons suitable for recording (resting membrane potential <−45 mV; access resistance <20 MΩ; capacitance <40 pF) were stimulated with 1-s current steps (−100 to +300 pA; 20 pA steps). AVP (Tocris) was diluted in extracellular solution and applied with a 5 μL Hamilton syringe. Dose-dependent responses (10^−12^-10^−6^ M) was generated to model physiological and supraphysiological levels in plasma and cerebrospinal fluid in humans and murine models.^6^^,^^11–13^ Data were manually analyzed using Clampfit 11.3 (Molecular Devices).

### Statistical analysis

Univariable datasets were compared by either a Wilcoxon (paired) or a Mann–Whitney (unpaired) *t*-test (2-tailed, α = .05). Repeat measurements were analyzed by 2-way ANOVA with Dunnett’s post hoc test. The effect of sex was assessed by 2-way ANOVA and Sidak’s post hoc test. All data presented as mean ± SEM. For each parameter and current step, values were pooled from ≥5 cells.

## Results

### Vasopressin increases excitability of rat renal dorsal root ganglia neurons

Measurement of AP generation in response to AVP (10^−12^-10^−6^ M) revealed a dose-dependent increase (*P* < .05) in mean AP frequency in rDRGN from both male SD rats (Figure 1A) and female (Figure 1B). A minimum AVP concentration of 10^−10^ M was required to elicit an effect in females, and 10^−8^ M in males. In male rDRGs, peak AP frequency was achieved with 10^−6^ M AVP (*P* < .05). In female rDRGs, peak AP frequency was achieved with 10^−10^ M AVP (*P* < .05).

**Figure 1:**
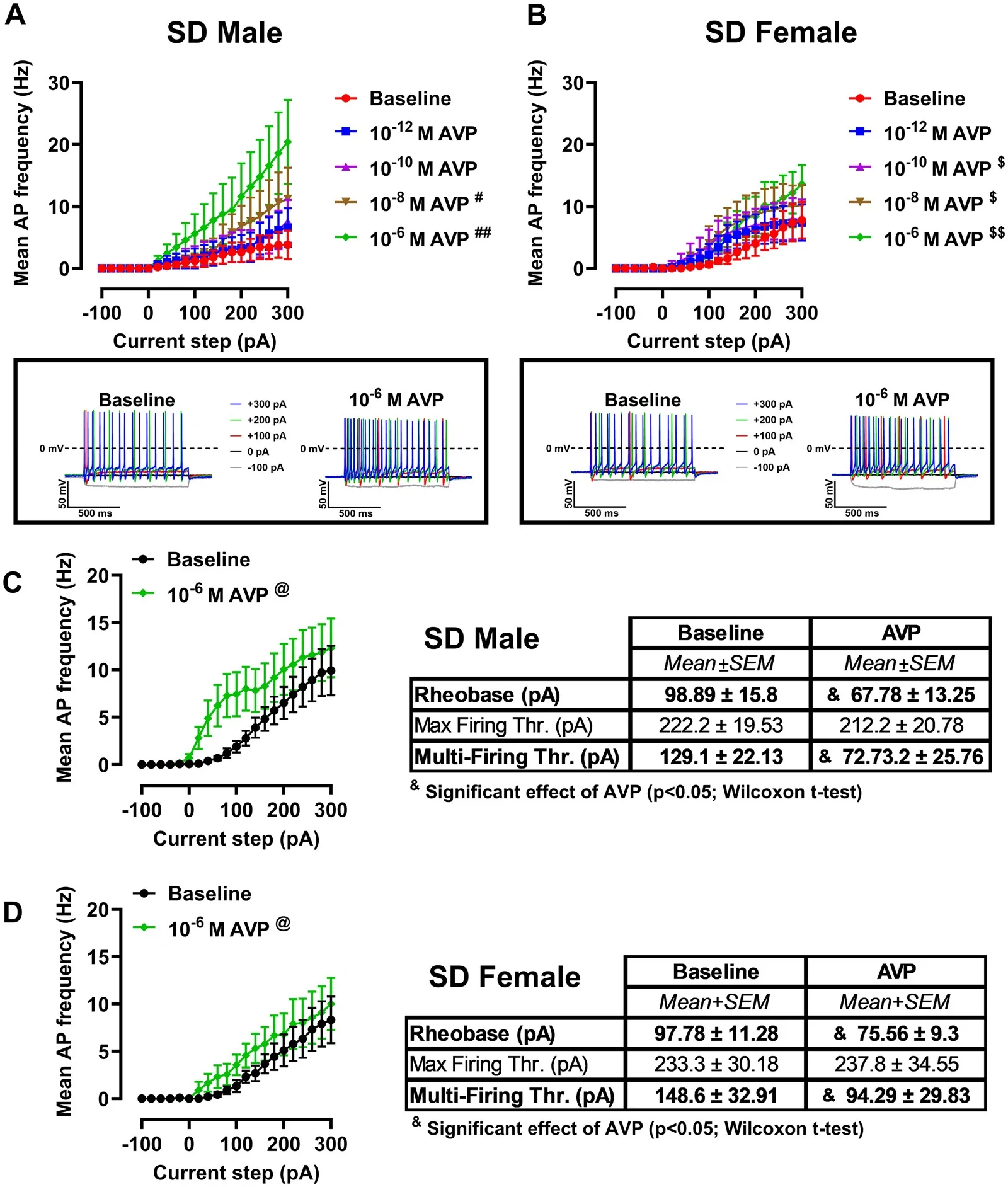
AVP Increases mean action potential frequency (AP_freq_) in isolated renal dorsal root ganglia neurons (rDRGNs). Dose-response curve shows increased AP_freq_ with increasing concentration of vasopressin (AVP) in both male (A) and female (B) rDRGNs (*n* = 5 cells from 3 male rats; *n* = 5 cells from 4 female rats). Representative AP_freq_ are shown in male (left) and female (right) rDRGNs before (baseline) and after AVP (10^−6^ M) application. Only current steps −100, 0, 100, 200, and 300 pA are displayed for clarity. Further measurements of responses to AVP (10^−6^ M) on AP_freq_ in male (C) and female (D) rat isolated rDRGNs. Tables show AVP effects on the mean rheobase, maximum firing threshold, and multi-firing threshold parameters in male (left) and female (right) rDRGNs (*n* = 18 cells from 8 male rats; *n* = 9 cells from 5 female rats). #: *P* < .05 vs any other group except 10^−10^ M (2-way ANOVA). ##: *P* < .05 vs any other group (2-way ANOVA). $: *P* < .05 vs baseline (2-way ANOVA). $$: *P* < .05 vs baseline and 10^−12^ M (2-way ANOVA). @: *P* < .05 vs baseline (2-way ANOVA). &: *P* < .05 vs baseline (Wilcoxon *t*-test).

Additional recordings in a subset of male and female rDRGNs (*n* ≥ 9 cells/group) were conducted to test 10^−6^ M AVP effects across sex. Mean AP frequency increased with AVP vs baseline (*P* < .05) in both male (*n* = 18; Figure 1C) and female (*n* = 9; Figure 1D) rDRGNs. No sex differences in mean AP frequency were detected at baseline (*P* > .05), but males showed an increased AP frequency with AVP compared to the females (*P* < .05). The rheobase, the current threshold for AP generation, was lower (*P* < .05) following AVP application, in both male (Figure 1C) and female (Figure 1D). No effect of AVP was detected on maximal firing threshold (current of highest AP generation); however, the multi-firing threshold (current threshold of >1 AP) was decreased with AVP (*P* < .05) in both male and female cells. No sex differences were detected in rheobase, maximal firing threshold, and multi-firing threshold. Importantly, no main effects of AVP or sex were detected on resting membrane potential (male: −52.63 ± 1.15 mV vs −52.37 ± 1.86 mV; female: −52.90 ± 2.87 mV vs −53.87 ± 2.76 mV).

## Conclusions

These data support our initial hypothesis, where AVP increased excitability of rDRGN from male and female SD rats. There was a clear sex difference in the response and sensitivity to AVP. Since the overall mean AP frequency response to AVP was higher in male rDRGN compared to female, this marks an increased overall AVP responsiveness in males vs females. Whether this difference is driven by chromosomal or hormonal mechanisms remains unknown, and will be investigated in future experiments.

Considering these sex differences, the AVP responses may partially explain the exacerbated phenotype and resting ARNA in males observed in our previous PKD studies,^3^ as well as the known sex differences in DOCA-salt HTN.^14^ Indeed, targeting renal sensory nerves by surgical ablation (ie, renal denervation) may be an effective way to interrupt this pathway. Yet, these data also highlight that targeting sensory interaction with AVP may also serve as an alternative pathway to treat cardiovascular and kidney diseases with elevated AVP. Notably, AVP reportedly drives both antinociceptive or pronociceptive effects at low or high concentrations, respectively.^15^ Thus, the high circulating AVP in patients suffering from hypertension^6^ and PKD^7^ might contribute to the increased afferent renal nerve activity and excitability.

A limitation of these experiments is the use of supraphysiological levels of AVP to determine sex-specific effects of AVP on rDRG electrophysiologic properties. It also remains unclear which AVP receptor(s), either V1a, V1b, or V2, mediates these effects. Based on recent human and mouse DRG transcriptomics, these neurons primarily express V1a and V2.^8^ While these data do not include rat samples, we speculate that the effects are driven through V1a–a G_q_-coupled receptor. Finally, recording from isolated DRG cell bodies does not permit the delineation between the effects of the cell body vs axonal/terminal interactions with AVP. Further experiments are planned to interrogate both the receptor-mediated mechanism and the central vs peripheral effects of AVP changes on renal afferent nerve activation.

## Data Availability

The data underlying this article will be shared on reasonable request to the corresponding author.
